# Self-supervised representation learning reveals explainable physiological structure in high-dimensional magnetocardiography

**DOI:** 10.1038/s41746-026-02819-8

**Published:** 2026-06-01

**Authors:** Dominik D. Kranz, Oruç Kahriman, Dominic Dischl, Sascha Treskatsch, André Sander, Johannes Brachmann, Jai-Wun Park, Niels Wessel

**Affiliations:** 1 Berlin Institute of Health, Berlin, Germany; 2 Charité – Universitätsmedizin Berlin, Berlin, Germany; 3 Department of Physics, Humboldt-Universität zu Berlin, Berlin, Germany; 4 ID Information und Dokumentation im Gesundheitswesen GmbH & Co. KGaA, Berlin, Germany; 5 German Heart Centre Munich, Munich, Germany; 6 Charité – Universitätsmedizin Berlin, Corporate Member of Freie Universität Berlin, Humboldt-Universität zu Berlin, and Berlin Institute of Health, Department of Anesthesiology and Intensive Care Medicine, Campus Benjamin Franklin, Berlin, Germany; 7 Sana Medical School, Coburg, Germany; 8 University of Split School of Medicine, Split, Croatia; 9 German Heart Center, DHZC at Charité – Universitätsmedizin Berlin, Berlin, Germany; 10 MSB Medical School Berlin, Berlin, Germany

**Keywords:** Cardiology, Computational biology and bioinformatics, Engineering

## Abstract

Artificial intelligence (AI) has shown strong performance in cardiology, but most approaches rely on sensing modalities whose physical limitations constrain available information. Magnetocardiography (MCG) records the cardiac magnetic field with less tissue distortion than surface potentials and may preserve higher-dimensional spatiotemporal electrophysiological structure. Here, we investigated whether combining MCG with self-supervised learning enables physiologically meaningful cardiac representations. We developed MCG2Vec, a contrastive encoder trained directly on raw 64-channel MCG recordings. Using recordings from 1732 consecutive patients, learned embeddings were evaluated with task-specific probes for multivessel coronary artery disease, reduced left ventricular ejection fraction, and paroxysmal atrial fibrillation risk from sinus-rhythm recordings. The representations enabled discrimination of multivessel coronary artery disease (area under the receiver operating characteristic curve (AUC) 0.89), reduced left ventricular ejection fraction (AUC 0.81), and atrial fibrillation risk (AUC 0.77). Attribution analyses revealed probe-specific temporal and spatial patterns corresponding to ventricular depolarization, repolarization, atrial activation dynamics, and coronary territories, supporting physiological interpretability. These findings suggest that higher-fidelity sensing combined with self-supervised representation learning can yield structured and explainable embeddings from non-invasive cardiac magnetic field recordings. More broadly, the study highlights measurement physics as an important determinant of what medical AI systems can learn.

## Introduction

Cardiovascular disease remains the leading cause of morbidity and mortality worldwide, underscoring the need for accurate, accessible, and non-invasive diagnostic tools capable of identifying disease at an early, potentially reversible stage^1–3^. Despite major technological advances, current cardiovascular diagnostics still rely on a trade-off between simplicity and precision^4^. At one end, electrocardiography (ECG) remains the global first-line tool due to its ubiquity and low cost^4^. However, the ECG records surface electrical potentials rather than the true three-dimensional myocardial electrical field^5^, limiting sensitivity and specificity for ischaemia, arrhythmogenic substrates, and subtle myocardial dysfunction^4,6^.

At the opposite end of the diagnostic spectrum, techniques such as coronary angiography, CT angiography, and nuclear perfusion imaging deliver detailed anatomical or perfusion information but involve radiation exposure, contrast agents, procedural risks, and limited accessibility^4,7^. Even cardiac MRI, while non-invasive and radiation-free, remains resource-intensive and typically limited to tertiary centres^8^. This leaves a diagnostic gap between inexpensive but coarse tools and high-fidelity, invasive, or resource-heavy modalities. In this gap, scalable digital sensing technologies capable of capturing rich electrophysiological information remain limited^9,10^.

Magnetocardiography (MCG) offers a biophysically motivated opportunity to narrow this gap. By recording the magnetic fields generated by myocardial electrical currents, MCG samples the cardiac electromagnetic field in a manner that is less affected by conductive tissue pathways than surface potentials^9–11^. In principle, this enables higher-fidelity access to the spatiotemporal structure of cardiac electrophysiology while retaining practical advantages of a non-invasive, contactless measurement. Despite these advantages, broader adoption has been constrained by the analytical challenges posed by high-dimensional, multichannel field recordings, particularly at scale.

The recent success of AI in cardiology has largely been driven by increasingly powerful models applied to fundamentally unchanged measurement modalities. This raises a critical question: to what extent are current performance limits imposed by model capacity, versus the biophysical constraints of the input signal itself? For high-dimensional biosignals such as MCG, a key challenge is to learn generalizable representations that capture stable morphology and field structure, while remaining robust to noise, missing channels, and acquisition variability.

Recent advances in self-supervised and contrastive learning provide a path to systematically explore the MCG signal space by learning generalizable representations directly from raw recordings^12–14^. These approaches offer the potential to extract subtle, physiologically relevant information that conventional analysis has been unable to exploit^15,16^.

Here, we aimed to develop an AI-based framework for analysing high-dimensional MCG recordings using self-supervised, contrastive representation learning. We pretrained an encoder directly on raw multichannel MCG signals and used complementary clinical labels as structured physiological probes: detection of high-burden obstructive coronary artery disease, estimation of left ventricular ejection fraction (LVEF), and identification of atrial fibrillation (AF) risk from sinus-rhythm recordings. We hypothesized that combining higher-fidelity magnetic field measurements with self-supervised representation learning yields embeddings that capture clinically relevant cardiac physiology. These tasks serve as functional probes of the learned representation, interrogating sensitivity to localized repolarization heterogeneity, global ventricular dysfunction, and latent atrial substrate.

## Results

### Cohort composition and data availability

**Fig. 1:**
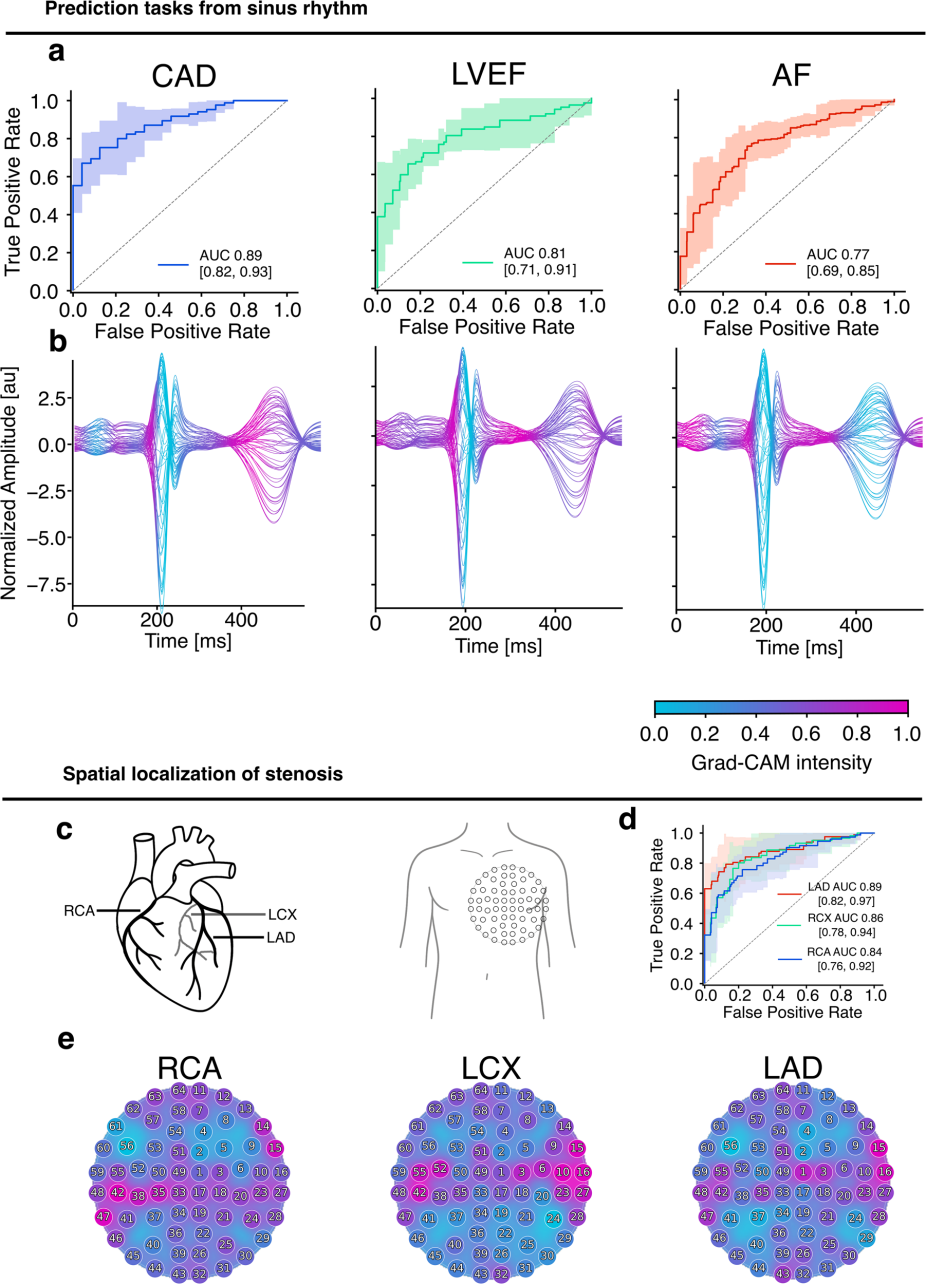
Learned magnetocardiographic representations encode complementary spatial and temporal cardiac physiology. **a** ROC curves for downstream probing of the learned MCG representations, targeting high-burden coronary artery disease (CAD), reduced left ventricular ejection fraction (LVEF), and paroxysmal atrial fibrillation (AF) risk from sinus-rhythm recordings. Shaded areas denote 95% bootstrapping confidence intervals. **b** Beat-aligned temporal importance profiles derived from Gradient-weighted Class Activation Mapping (Grad-CAM), averaged across validation cohorts. Colour intensity indicates relative contribution of each time point to the corresponding probe, revealing probe-specific temporal signatures aligned with distinct phases of the cardiac cycle. **c** Schematic illustration of major coronary artery territories (left anterior descending artery, left circumflex artery, right coronary artery) and corresponding sensor array placement over the anterior thorax. **d** ROC curves for vessel-specific stenosis probing, demonstrating consistent performance across coronary territories. **e** Spatial projection of Grad-CAM importance scores onto the 64-channel MCG sensor array for each coronary territory. Distinct topographic activation patterns indicate that the learned representations preserve spatial organization corresponding to underlying coronary anatomy.

From 1732 consecutive patients undergoing clinical magnetocardiography, task-specific cohorts were defined based on the availability of corresponding reference standards and signal quality (Fig. 1c). This resulted in cohorts of 208 patients for high-burden coronary artery disease (CAD) probing, 238 patients for left ventricular ejection fraction (LVEF) probing, and 454 patients with sinus-rhythm recordings for atrial fibrillation (AF) substrate probing. Detailed cohort characteristics and clinical distributions are provided in Supplementary Table 1.

### Learned representations support complementary downstream probes

To assess which aspects of cardiac physiology are encoded in the learned magnetocardiographic representations, the pretrained MCG2Vec encoder was coupled to task-specific probing heads targeting complementary diagnostic domains.

**Fig. 2:**
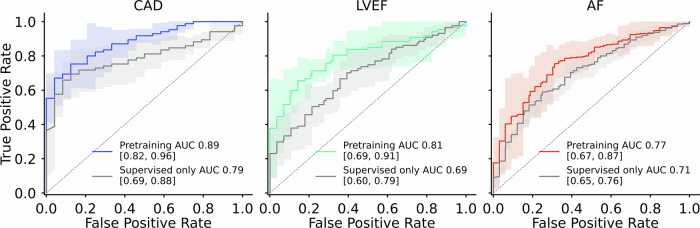
Self-supervised pretraining improves MCG-based downstream classification. ROC curves for three downstream probes, high-burden CAD, AF risk from sinus-rhythm recordings, and reduced LVEF, comparing a contrastively pretrained MCG2Vec encoder followed by task-specific probing (coloured curves) versus training the same encoder–head architecture end-to-end using supervised objectives only (grey curves). Shaded regions indicate 95% confidence intervals across patient-stratified cross-validation folds. Across all tasks, self-supervised pretraining yields higher AUCs, consistent with improved robustness to acquisition variability and a bias toward morphology-based representations.

When probed for high-burden obstructive CAD, the learned representations enabled reliable discrimination between patients with multivessel stenosis and those without (mean AUC 0.89, 95% CI 0.82–0.93; Fig. 2a). Probing for reduced systolic function yielded a mean AUC of 0.81 (95% CI 0.71–0.91), indicating that global ventricular performance is reflected in the magnetic field representations. Probing for paroxysmal AF risk from sinus-rhythm recordings achieved a mean AUC of 0.77 (95% CI 0.69–0.85), despite the absence of active arrhythmia during acquisition.

Together, these results demonstrate that a single self-supervised representation learned from raw MCG signals supports multiple downstream probes targeting spatially localized pathology, global myocardial function, and latent electrophysiological instability. A detailed summary of patient-level validation metrics, including sensitivity, specificity, accuracy, precision, and F1-score, is provided in Table 1.

**Table 1:** Patient-level validation performance across downstream tasks. Values are mean (95% CI) across folds

Task	AUC	Sensitivity	Specificity	Accuracy	Precision	F1-score
AF risk	0.77 (0.69, 0.85)	0.70 (0.63, 0.75)	0.74 (0.67, 0.80)	0.73 (0.65, 0.78)	0.79 (0.76, 0.83)	0.72 (0.63, 0.79)
Reduced LVEF	0.81 (0.71, 0.91)	0.70 (0.63, 0.78)	0.77 (0.69, 0.84)	0.74 (0.67, 0.80)	0.73 (0.66, 0.80)	0.71 (0.64, 0.78)
Multivessel CAD	0.89 (0.82, 0.97)	0.77 (0.71, 0.81)	0.84 (0.79, 0.88)	0.81 (0.77, 0.84)	0.90 (0.86, 0.92)	0.83 (0.80, 0.85)

### Coronary anatomy is preserved in representations

To further interrogate whether spatial information is preserved in the learned embeddings, the encoder was probed for vessel-specific obstructive stenosis. Performance remained consistent across coronary territories, with AUCs of 0.89 (95% CI 0.82–0.97) for the left anterior descending artery (LAD), 0.86 (95% CI 0.78–0.94) for the left circumflex artery (LCX), and 0.84 (95% CI 0.76–0.92) for the right coronary artery (RCA) (Fig. 2d). A corresponding summary of patient-level performance metrics for vessel-specific localization is provided in Table 2.

**Table 2:** Patient-level validation performance for vessel-specific localization. Values are mean (95% CI) across folds

Task	AUC	Sensitivity	Specificity	Accuracy	Precision	F1-score
LAD	0.89 (0.82, 0.97)	0.78 (0.65, 0.87)	0.88 (0.83, 0.92)	0.84 (0.77, 0.90)	0.85 (0.81, 0.89)	0.81 (0.71, 0.89)
RCA	0.84 (0.76, 0.92)	0.68 (0.64, 0.74)	0.79 (0.74, 0.84)	0.73 (0.69, 0.77)	0.76 (0.70, 0.81)	0.72 (0.68, 0.76)
LCX	0.86 (0.78, 0.94)	0.68 (0.60, 0.74)	0.78 (0.73, 0.84)	0.73 (0.67, 0.78)	0.77 (0.71, 0.82)	0.72 (0.66, 0.79)
Micro-average	0.83 (0.76, 0.89)	0.72 (0.64, 0.77)	0.81 (0.77, 0.84)	0.75 (0.71, 0.79)	0.75 (0.69, 0.80)	0.73 (0.67, 0.78)

Projection of importance scores derived from Gradient-weighted Class Activation (Grad-CAM^17^) onto the 64-channel sensor array revealed distinct topographic activation patterns associated with each vascular territory (Fig. 2e). These vessel-specific spatial signatures indicate that the learned representations retain spatial organization corresponding to underlying coronary anatomy, rather than collapsing information into a purely global descriptor.

### Temporal structure aligns with cardiac electrophysiology

To evaluate the physiological plausibility of the learned representations, temporal importance profiles were extracted using Grad-CAM and aligned to the cardiac cycle. Beat-averaged importance maps revealed probe-specific temporal signatures (Fig. 2b).

For CAD probing, salient contributions were distributed across both early depolarization and the ST–T segment, consistent with spatially heterogeneous repolarization dynamics. In contrast, probing for reduced LVEF emphasized the ventricular depolarization phase, centring on the onset and peak of the QRS complex. AF risk probing exhibited a distinct temporal focus involving the P wave and terminal QRS components, consistent with latent atrial substrate and conduction abnormalities.

These findings demonstrate that the learned representations encode temporally distinct features aligned with established electrophysiological processes, rather than nonspecific waveform characteristics.

### Self-supervised pretraining improves generalization compared with end-to-end supervised training

**Fig. 3:**
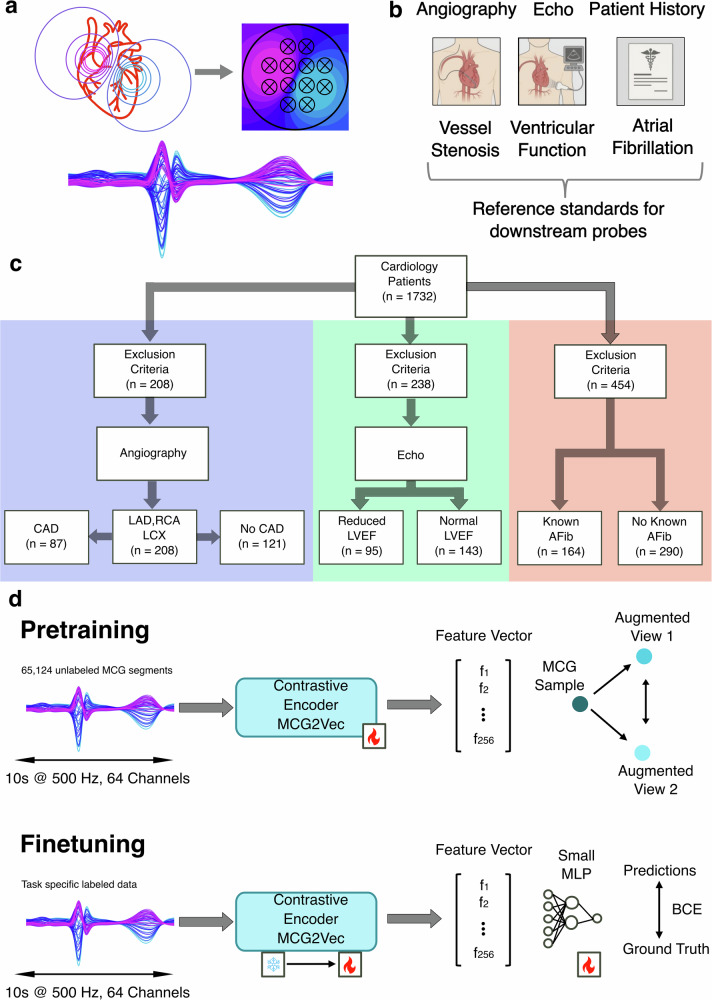
Overview of magnetocardiography acquisition, self-supervised representation learning, and downstream probing. **a** Biophysical principle of MCG. Cardiac electrical activity generates magnetic fields that are sampled non-invasively by a spatially resolved 64-channel SQUID sensor array positioned above the anterior thorax, yielding multichannel cardiac magnetic field waveforms. **b** Reference standards used for downstream probes. MCG recordings were paired with complementary clinical reference modalities—coronary angiography, transthoracic echocardiography, and structured patient history—to derive labels reflecting vessel-level stenosis, ventricular function, and atrial fibrillation status, respectively. **c** Cohort construction and task-specific data availability. From 1732 consecutive cardiology patients, task-specific cohorts were defined based on the availability of corresponding reference standards, following predefined exclusion criteria. **d** Self-supervised representation learning and downstream probing. During pretraining, unlabelled 10-s MCG segments (64 channels, 500 Hz) were encoded using a contrastive learning framework (MCG2Vec) that maximizes agreement between augmented views of the same signal. The pretrained encoder was subsequently coupled to shallow task-specific probing heads and fine-tuned using labelled data to interrogate which diagnostic information is encoded in the learned magnetic field representations. Panels are ordered to reflect the experimental pipeline from biophysical measurement to learned representations and downstream probes. Abbreviations: MLP, multilayer perceptron; BCE, binary cross-entropy loss.

Using the same encoder–classifier architecture, we compared end-to-end supervised training with a self-supervised pretraining followed by task-specific probing. Across all three downstream probes, self-supervised pretraining improved patient-level discrimination relative to end-to-end supervised training (CAD: AUC 0.89 vs 0.79; LVEF: 0.81 vs 0.69; AF: 0.77 vs 0.71; Fig. 3). These differences suggest that contrastive objectives and strong augmentations bias learning toward morphology-based features that generalize across patients and acquisition variability, whereas end-to-end supervision is more susceptible to exploiting acquisition-specific correlates.

### Summary of representational findings

Across all downstream probes, the pretrained MCG2Vec encoder consistently supported discrimination of complementary cardiac phenotypes while preserving spatial and temporal structure that aligns with known cardiac physiology. Performance metrics across tasks (Tables 1, 2) provide quantitative evidence for the diagnostic relevance of these representations, while spatial and temporal analyses indicate that the learned embeddings retain physiologically meaningful organization at both the field and waveform level.

## Discussion

This study demonstrates that self-supervised representation learning applied to high-dimensional MCG yields latent embeddings that encode spatially and temporally structured cardiac physiology from short, sinus-rhythm recordings. A single pretrained encoder supported downstream probes spanning localized coronary pathology, global ventricular function, and latent atrial electrophysiological instability, indicating that physiologically and clinically relevant information across distinct pathophysiological domains is jointly represented in the cardiac magnetic field.

A central implication of these findings is that the performance of AI systems in cardiology is fundamentally shaped by the physical properties of the sensing modality. While recent work has shown that deep learning can extract clinically useful correlates from surface ECG^18–20^, such models operate on signals that are intrinsically attenuated and spatially smoothed by conductive tissue pathways. In contrast, magnetic fields generated by cardiac electrical currents propagate through tissue with minimal distortion, preserving higher-order spatiotemporal structure. Our results suggest that this increased physical fidelity may translate into representations that retain spatial organization and temporal specificity aligned with underlying electrophysiological processes, thereby expanding the space of physiological features accessible to data-driven models. Direct comparative evaluation against ECG-based models under matched conditions represents an important direction for future work.

The observed vessel-specific spatial signatures and probe-specific temporal activation patterns support this interpretation. Localization of obstructive stenosis to individual coronary territories indicates that the learned representations preserve spatial gradients in the magnetic field consistent with regional repolarization heterogeneity. Similarly, distinct temporal emphasis during depolarization, repolarization, or atrial activation across downstream probes suggests that the encoder captures physiologically meaningful dynamics rather than generic waveform features. We note that variations in chest-to-sensor (standoff) distance can influence signal amplitude across the array; however, the use of normalization and augmentation strategies reduces reliance on absolute amplitude, and the consistent vessel-specific patterns observed across subjects suggest that the model captures structured spatial information rather than purely distance-dependent effects. These findings align with theoretical and experimental work indicating that MCG is sensitive not only to transmembrane potentials but also to intracellular primary currents, which may amplify subtle electrophysiological alterations that remain subthreshold in surface potentials^21,22^. Importantly, these spatial and temporal correspondences provide an interpretable link between learned representations and established cardiac physiology.

More broadly, this work contributes to a growing body of evidence that advances in AI must be accompanied by advances in measurement to unlock their full potential^23^. Many prior studies in medical AI have focused on increasingly sophisticated models applied to unchanged sensing modalities, often evaluated on narrowly defined or convenience tasks^24,25^. Such designs risk conflating true physiological inference with proxy signals related to demographics, referral patterns, or disease prevalence^26,27^. By contrast, the present framework emphasizes representation learning from a biophysically motivated measurement and evaluates learned embeddings through multiple complementary probes, providing a principled approach to assessing information content rather than task-specific optimization alone.

Importantly, interpretability analyses revealed that the learned representations align with established cardiac electrophysiology, providing physiological validation beyond predictive performance. The correspondence between activation profiles and known phases of the cardiac cycle mitigates concerns that the model relies on spurious correlations or acquisition artefacts, a persistent barrier to trust and adoption in clinical AI systems. Here, explainability is achieved through physiologically grounded spatial and temporal attribution rather than post-hoc rationalization of model outputs. In this context, contrastive pretraining serves not only as a data-efficient learning strategy but also as a mechanism to enforce invariance to non-physiological variability while preserving meaningful morphology.

Beyond measurement fidelity, our findings highlight the role of representation learning objectives in determining whether deep learning models encode physiological structure or exploit non-generalizable shortcuts. In high-dimensional biosignals such as MCG, the space of acquisition-specific artefacts and session fingerprints can exceed the space of task-relevant physiological variation, particularly under limited supervision. Purely supervised objectives therefore, permit degenerate solutions that satisfy the loss without learning meaningful morphology.

Contrastive pretraining can be interpreted as imposing a structured constraint on the hypothesis space of the model. Rather than allowing arbitrary features that optimize a supervised objective, the contrastive formulation enforces invariance to transformations that disrupt acquisition-specific variability while preserving underlying signal morphology. In the context of MCG, where measurements reflect the superposition of spatially distributed cardiac current sources, these invariances selectively suppress features tied to sensor configuration, noise, or amplitude scaling, and promote features that are stable under biophysically plausible transformations of the electromagnetic field.

Under this perspective, the learned representation is biased toward encoding intrinsic electrophysiological structure. This is supported by the observed alignment of latent features with temporally distinct phases of the cardiac cycle and spatially localized activation patterns corresponding to coronary territories. Rather than individual latent dimensions mapping directly to specific physiological variables, the representation appears to organize into subspaces capturing complementary aspects of cardiac dynamics, including depolarization, repolarization, and spatial current distribution.

The choice of contrastive objective is also aligned with the nature of the signal. Unlike modalities where predictive temporal structure is the primary signal of interest, MCG encodes physiologically relevant information in the morphology and spatial organization of the cardiac magnetic field within individual cycles. Objectives such as Contrastive Predictive Encoding (CPC)^28^ are therefore less directly matched to this setting, while non-contrastive approaches such as “Bootstrap your own latents”^29^ may rely more heavily on implicit regularization to avoid collapse. By contrast, the instance-discriminative formulation of SimCLR, combined with explicitly designed augmentations, provides direct control over which transformations define equivalence, enabling the representation to be shaped by biophysically motivated invariances. Nevertheless, future work should systematically evaluate and compare different self-supervised learning strategies for high-dimensional electromagnetic field data, particularly with respect to their ability to capture physiologically meaningful structure.

Although this study focuses on cardiology, the implications extend beyond a single organ system. The combination of high-fidelity biophysical sensing with self-supervised representation learning provides a general framework for extracting latent physiological structure from complex biosignals. Similar principles may apply to other domains where measurement physics constrains downstream inference, including neurophysiology, bioelectromagnetism, and functional imaging.

Several limitations should be acknowledged. First, while the data was acquired prospectively, the AI development is retrospective and single-centre, and external validation across independent institutions and acquisition systems will be essential to assess generalizability. Second, atrial fibrillation status was derived from clinical history rather than continuous rhythm monitoring, introducing potential label noise, particularly in the control group. Third, a subset of recordings was acquired using manufacturer-provided hardware bandpass filtering, which may attenuate high-frequency magnetic field components; future studies using unfiltered acquisitions may further enhance representation fidelity. Finally, coronary artery disease was defined anatomically rather than functionally; integration with physiological reference standards such as fractional flow reserve or microvascular indices would provide deeper insight into structure–function relationships. Future work should integrate biophysical modelling approaches, such as simulation of cardiac magnetic fields under controlled conditions (e.g., coronary stenosis models), to further link learned representations to underlying electrophysiological mechanisms.

In summary, this work demonstrates that combining high-fidelity magnetocardiographic sensing with self-supervised representation learning yields latent embeddings that encode physiologically structured and explainable cardiac physiology across spatial and temporal scales. Rather than relying solely on increasingly complex models applied to physically constrained signals, this framework highlights the importance of measurement fidelity as a determinant of what AI systems can learn.

By revealing structured, physiologically grounded representations from non-invasive cardiac magnetic field recordings, this study positions magnetocardiography as a meaningful complement to established electrophysiological modalities and offers a framework for modality-aware artificial intelligence in biomedicine. Rather than establishing clinical readiness, these findings provide a foundation for future studies exploring how AI-enabled MCG may contribute to electrophysiological phenotyping under prospective and multi-site validation. If validated under such conditions, AI-enabled MCG could support earlier and more scalable electrophysiological phenotyping in routine care, ranging from risk stratification in chest-pain pathways to longitudinal monitoring of myocardial dysfunction and atrial substrate, thereby complementing imaging-heavy diagnostic cascades with a rapid, contactless assessment. The diagnostic utility of superconducting quantum interference device (SQUID)-based MCG in this study is enabled by high sensitivity, reproducible signal-to-noise characteristics, and controlled measurement conditions afforded by magnetic shielding. Extending these findings to unshielded or non-SQUID MCG systems will require dedicated validation under matched acquisition protocols and clinical reference standards. Emerging room-temperature and potentially unshielded MCG systems, particularly those based on optically pumped magnetometer (OPM) technology, represent a promising direction toward more accessible and scalable deployment. While low critical temperature (low-Tc) SQUID systems currently provide superior signal-to-noise characteristics under controlled conditions, we view these approaches as complementary: high-fidelity shielded recordings may serve as a reference for developing and validating models, which can subsequently be adapted to more portable acquisition settings. More broadly, the convergence of biophysical sensing and representation learning highlighted here points toward AI systems that are not only accurate but also interpretable, generalizable, and anchored in underlying physical principles.

Looking ahead, the future impact of MCG will depend on coordinated efforts to standardize acquisition, calibration, and evaluation across rapidly diversifying sensor technologies, including shielded SQUID systems and emerging OPM platforms operating under varying shielding and environmental noise conditions. Establishing community benchmarks, i.e., shared datasets with harmonized metadata, agreed-upon preprocessing and quality metrics, and matched clinical reference standards, would enable rigorous cross-system comparisons and clarify which aspects of cardiac magnetic field structure are robust to hardware and site variability. Such standardization will be essential for translating MCG from promising single-system demonstrations into a reproducible, clinical modality and for ensuring that representation learning models trained on one acquisition regime remain valid when deployed across different sensor configurations and clinical environments. At present, external validation across independent institutions is constrained by the limited availability of standardized, multi-center MCG datasets, as acquisition protocols, sensor configurations, and preprocessing pipelines vary substantially across sites. Importantly, we do not claim clinical readiness in the absence of such validation. Rather, this work is intended as a step toward establishing representation learning on high-fidelity cardiac magnetic field measurements, providing a foundation for future multi-center validation and clinical translation. In this context, and motivated by the need for comparability across studies, we propose a minimum reporting framework for future MCG research, summarized in Table 3.

**Table 3:** Proposed minimum reporting checklist for magnetocardiography studies

Domain	Item	Reporting requirement
**Instrumentation**	Sensor system	Specify sensor type (e.g., SQUID, OPM), number of channels, and system configuration
	Field components	Report measured magnetic field component(s) (e.g., Bz, Bx/By/Bz) and sensor orientation
**Measurement geometry**	Array layout	Provide sensor layout or channel map relative to the thorax
	Standoff distance	Report the chest-to-sensor distance and its variability across subjects
	Positioning	Describe patient positioning and alignment (e.g., anatomical landmarks, intercostal placement)
**Acquisition environment**	Shielding	Specify shielding conditions (shielded, partially shielded, unshielded)
	Noise conditions	Report sampling rate, filtering, and relevant noise characteristics or suppression methods
**Physiological context**	Recording protocol	Report recording duration, posture, and physiological state (e.g., sinus rhythm, rest/stress)
	Synchronization	Specify ECG co-recording and alignment or gating strategy if used
**Preprocessing**	Signal processing	Describe filtering, artefact handling, segmentation, and normalization steps
**Data representation**	Signal format	Specify whether the analysis uses raw signals, averaged beats, maps, or derived representations
**Clinical reference**	Labels	Define clinical endpoints and reference standards (e.g., angiography, imaging, clinical history)
**Evaluation**	Validation design	Report cohort construction, patient-level separation, and validation strategy

## Methods

### Study design and population

A total of 1732 consecutive patients undergoing prospectively acquired clinical MCG examination were included. Recordings were acquired at the Cardiology Department of Asklepios Hospital Hamburg, Germany, following a predefined acquisition protocol as part of an ambulatory evaluation for suspected cardiovascular disease. For the model development, patients were eligible if a technically adequate MCG recording was available and at least one corresponding clinical reference standard existed for downstream analyses. Exclusion criteria comprised acute coronary syndrome, insufficient signal quality, incomplete documentation, or missing reference data. Task-specific cohorts were defined based on the availability of corresponding clinical reference standards (e.g., multivessel CAD, reduced LVEF, AF status), as detailed in Supplementary Table 1. The study reflects a real-world clinical population, in which patients may present with comorbidities and prior treatments; no attempt was made to isolate single-disease cohorts. While this may introduce potential confounding factors, it reflects the real-world clinical population encountered in routine cardiology practice. The study was approved by the Ethics Committee of the Medical Association Hamburg (Ärztekammer Hamburg; approval number PV4552). The study was conducted in accordance with the Declaration of Helsinki. All participants provided written informed consent prior to participation.

### MCG acquisition and preprocessing

**Fig. 4:**
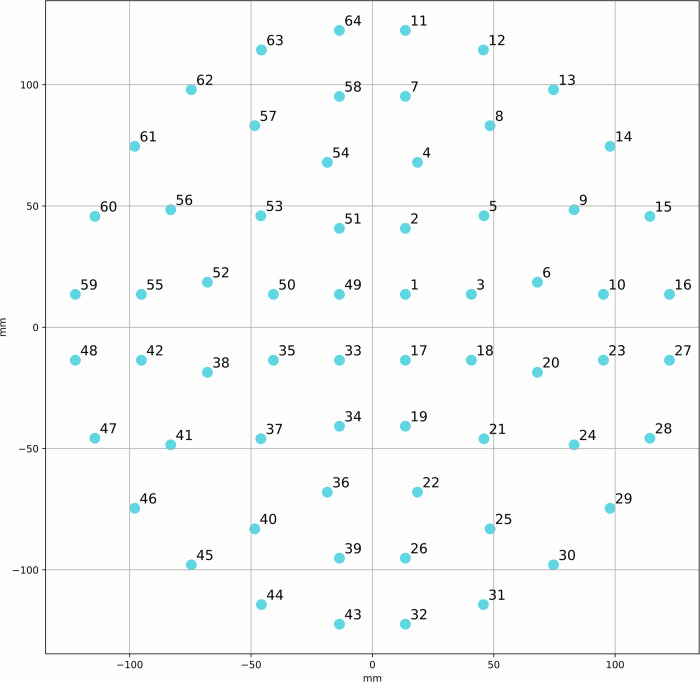
Sensor configuration of the MCG measurement system. All sensors measured the normal component of the magnetic field (Bz).

MCG recordings were obtained using a 64-channel, second-generation low-Tc superconducting quantum interference device (SQUID) system (CS-MAG II, Biomagnetik Park GmbH, Germany), which measures the normal component (Bz) of the cardiac magnetic field. Sensors were arranged in a fixed circular grid positioned above the anterior thorax with the patient in supine position, enabling spatially resolved sampling of the cardiac magnetic field. The typical chest-to-sensor (standoff) distance was approximately 1 cm, with minor variation across subjects due to anatomical differences and positioning. A sensor diagram containing exact measurements can be found Fig. 4. Recordings were performed in a magnetically shielded environment at a sampling rate of 500 Hz using manufacturer-provided hardware band-pass filtering. Each acquisition consisted of several minutes of continuous recording following a standardized protocol.

### Signal preprocessing and quality control

Continuous MCG recordings were segmented into non-overlapping 10-second windows at the native sampling rate (64 channels × 5000 samples). To preserve spatial sensor geometry, channels missing due to technical issues were retained as zero-valued traces at their respective array positions. Signals underwent standardized preprocessing, including a fifth-order Butterworth high-pass filter with a cutoff frequency of 0.5 Hz to remove baseline drift and a notch filter at 50 Hz to suppress power-line interference, applied in zero-phase configuration to preserve waveform morphology. To estimate signal quality, we employed a data-driven signal-to-noise ratio (SNR) measure based on the geometric regularity of cardiac cycles. Specifically, MCG segments were aligned around detected cardiac cycles, and a low-rank reconstruction was obtained using singular value decomposition (SVD). The noise component was defined as the residual between the original signal and its low-rank reconstruction, and SNR was computed as the logarithmic ratio of signal to noise power. This approach relies on the assumption that clean cardiac cycles can be represented by a small number of dominant components, while noise is captured in higher-order residuals, and has been previously described in the context of cardiac biosignal analysis^30^. Segments with a mean SNR < 5 were excluded. This threshold was determined empirically based on visual inspection by an experienced clinician to ensure adequate signal quality.

### MCG2Vec encoder architecture

We implemented a convolutional encoder (MCG2Vec) designed to learn compact representations from high-dimensional multichannel MCG signals. The architecture operates directly on raw 64-channel time series, using temporal convolutions to capture local waveform morphology while preserving inter-channel structure inherent to the sensor array. The encoder maps each 10-second segment to a fixed-dimensional latent embedding.

### Contrastive pretraining objective

The encoder was pretrained using a SimCLR-style contrastive learning framework^31^. For each MCG segment, two independently augmented views were generated and passed through the shared encoder. Augmentations were selected to reflect plausible non-physiological variability while preserving underlying cardiac dynamics: (i) additive Gaussian noise scaled to the per-channel standard deviation, (ii) random channel dropout in which 10% of channels were replaced by noise drawn from their own distribution, and (iii) per-channel temporal masking, where a contiguous portion of up to approximately 75% of the segment was replaced by the channel mean. This temporal masking may result in augmented views containing partially non-overlapping cardiac cycles, thereby encouraging the model to identify consistent spatiotemporal field structure across repeated realizations of the same underlying cardiac dynamics, rather than relying on cycle-specific features. Augmentation strengths were intentionally chosen to be relatively high, such that the contrastive task requires matching signals under substantial corruption, thereby encouraging the model to rely on robust morphological and spatial structure rather than superficial or acquisition-specific features. The network was optimized using the NT-Xent loss with a temperature of 0.1, encouraging invariance to channel-specific noise, missing-sensor patterns, and local signal corruption. These augmentations were chosen to approximate transformations that alter measurement conditions without changing the underlying cardiac electromagnetic field. Augmentation parameters were determined based on domain knowledge and empirical tuning. A systematic ablation study of augmentation strategies was not performed and represents an important direction for future work.

### Rationale for contrastive formulation

We adopt an instance-discriminative contrastive objective (SimCLR) due to its alignment with the structure of MCG signals. In contrast to predictive objectives that emphasize temporal forecasting, the primary objective in MCG is to capture stable spatiotemporal field morphology within individual cardiac cycles. SimCLR directly optimizes for invariance across augmented views of the same segment, thereby encouraging representations that are robust to acquisition variability while preserving morphology.

Alternative self-supervised approaches, such as BYOL, remove the need for negative samples but may admit representational collapse without careful regularization, particularly in relatively homogeneous biomedical signals. Predictive frameworks such as CPC are designed to model temporal dependencies, but place less emphasis on spatial field structure and morphology. In contrast, the SimCLR objective naturally accommodates multichannel inputs and allows explicit control over invariances through augmentation design, making it well-suited for high-dimensional MCG signals.

### Contrastive objective and biophysical interpretation

The SimCLR objective enforces that different augmented views of the same MCG segment are mapped to similar representations, while representations of different segments are separated. In this work, augmentations are designed to selectively perturb acquisition-dependent variability, including sensor noise, amplitude scaling, and partial channel dropout, while preserving the underlying spatiotemporal structure of the cardiac magnetic field.

Under this formulation, instance identity corresponds to a specific realization of cardiac electromagnetic activity, governed by spatially distributed current sources and their temporal dynamics. By enforcing invariance to transformations that do not alter this underlying field structure, the contrastive objective biases the encoder toward features that are stable under biophysically plausible perturbations.

Given that the cardiac magnetic field reflects the superposition of primary and volume currents, this encourages the representation to encode consistent aspects of depolarization, repolarization, and spatial current distribution, while suppressing features tied to acquisition-specific variability.

### Optimization and training

Pretraining was performed for 100 epochs using data-parallel training across multiple GPUs with a global batch size of 32 × number of GPUs. Optimization employed AdamW^32^ with an initial learning rate of 1 × 10^-3^, cosine decay over the full training schedule, final learning rate of 1% of the initial value, and weight decay of 1 × 10^-4^. The pretrained encoder weights were retained for all downstream analyses.

### Downstream probing task definitions and label construction

Three downstream tasks were defined to probe complementary aspects of the learned MCG representations. For coronary artery disease (CAD), stenosis severity was available as binary per-vessel indicators (LAD, RCA, LCX; ≥70% vs <70%). To reduce label ambiguity, high-burden obstructive CAD was defined as ≥70% stenosis in at least two major epicardial vessels. Vessel-specific labels were used for spatial localization analyses. For systolic function, reduced left ventricular ejection fraction (LVEF) was defined as <50% based on transthoracic echocardiography performed within a clinically accepted temporal window. For arrhythmic substrate probing, paroxysmal atrial fibrillation (AF) status was determined from structured clinical records. Only sinus-rhythm MCG recordings were included; recordings with active AF were excluded.

### Probing heads and fine-tuning

For each task, a shallow task-specific classifier was attached to the pretrained encoder. All downstream tasks were evaluated using a shared pretrained encoder, with task-specific probing heads trained independently for each clinical endpoint. No task-specific modification of the encoder architecture was performed. Ten-second MCG segments served as input, and the encoder output was passed through dropout (0.2), a 64-unit fully connected layer with swish activation, and a final sigmoid output neuron. Fine-tuning was performed using patient-level stratified five-fold cross-validation to prevent information leakage across splits. Each fold began with a five-epoch warm-up phase with the encoder frozen, followed by joint optimization of encoder and classifier parameters for 30 epochs. Binary cross-entropy loss with class-frequency-weighted samples was minimized using Adam.

### Evaluation protocol

Cross-validation was performed at the patient level, such that all MCG segments from a given patient were assigned exclusively to a single fold, ensuring strict separation between training and validation data and preventing information leakage.

Model performance was quantified using AUC, with confidence intervals computed across cross-validation folds. Where applicable, segment-level predictions were aggregated at the patient level.

### Physiological validation and interpretability

To assess whether learned representations captured physiologically meaningful signal features, we applied Gradient-weighted Class Activation Mapping (Grad-CAM)^17^ to generate temporal importance profiles for each MCG segment. Importance maps were aligned to R-peaks and averaged across validation cohorts to obtain beat-resolved attention profiles. This enabled inspection of which phases of the cardiac cycle contributed most strongly to model outputs. In addition, channel-wise importance scores were projected onto the sensor array to examine spatial patterns associated with different downstream probes. These analyses were used to verify correspondence between learned features and established electrophysiological processes, including depolarization, repolarization, and atrial remodelling.

During the preparation of this manuscript, generative AI tools (ChatGPT, OpenAI) were used exclusively for linguistic editing of the final text. All scientific content, analysis, and interpretation were developed and verified by the authors, who take full responsibility for the manuscript.

## Supplementary information

Supplementary Information

## Data Availability

The datasets generated during the current study are not publicly available due to patient privacy, institutional data protection regulations, and consent restrictions, but are available from the corresponding author on reasonable request.

## Code availability

Custom code used for model development, preprocessing, training, and statistical analysis will be made publicly available upon publication under https://github.com/KranzDD. Architectural details and key training parameters are described in the Methods.
