# Supplementary material for: Self-supervised representation learning reveals explainable physiological structure in high-dimensional magnetocardiography
Source: NPJ Digit Med. 2026 Jun 1;9:412. doi: 10.1038/s41746-026-02819-8 (PMC13226652; doi:10.1038/s41746-026-02819-8)
Supplement: Supplementary file 1 — Supplementary Information [file 41746_2026_2819_MOESM1_ESM.pdf]

Supplementary Table 1 Clinical cohort description. Statistically significant ($p < 0.05$) distributional differences in bold (Mann-Whitney-U Test for continuous variables, χ^2 for categorical variables).

**Clinical Task**	**CAD**		**Localization**			**LVEF**		**AF**	
Positive Definition	> 70% stenosis in at least 2 major vessels (LAD, RCA, LCX)		> 70% Stenosis in LAD, RCA, LCX (multilabel possible)			LVEF < 50%		Known patient history of AF	
Negative Definition	< 70% Stenosis in all three vessels		< 70% Stenosis in LAD, RCA, LCX (multilabel possible)			LVEF > 50%		No known patient history of AF	
Cohort Feature	Pos	Neg	LAD Pos	RCA Pos	LCX Pos	Pos	Neg	Pos	Neg
Number	87	121	81	71	61	95	143	164	290
Age (y)	**71.82 ± 10.73**	**63.84 ± 15.21**	72.05 ± 10.76	71.64 ± 10.67	73.39 ± 9.74	**69.85 ± 12.00**	**65.34 ± 14.09**	**71.26 ± 9.80**	**65.28 ± 14.29**
BMI (kg /m)	27.58 ± 4.77	27.30 ± 4.56	27.63 ± 4.78	27.15 ± 4.38	27.81 ± 4.51	26.57 ± 4.39	27.57 ± 5.02	28.05 ± 5.63	27.12 ± 4.77
Sex	**65m 22f ***	**51m, 70f ***	63m 18f	53m 18f	48m 13f	**66m, 29f***	**77m, 66f***	88m 78f	175m 123f
Acute Chest Pain	17%	22%	15%	18%	14%	14%	22%	**12%***	**20%***
Art. Hyperten sion	100%	100%	100%	100%	100%	100%	100%	100%	100%
